# Association between serum insulin-like growth factor I or IGF-binding protein 3 and estimated glomerular filtration rate: results of a population-based sample

**DOI:** 10.1186/1471-2369-13-169

**Published:** 2012-12-13

**Authors:** Kathleen Dittmann, Henri Wallaschofski, Rainer Rettig, Sylvia Stracke, Karlhans Endlich, Henry Völzke, Matthias Nauck, Nele Friedrich

**Affiliations:** 1 Institute of Clinical Chemistry and Laboratory Medicine, Ernst-Moritz-Arndt University of Greifswald, Greifswald, Germany; 2 Institute of Physiology, Ernst-Moritz-Arndt University of Greifswald, Greifswald, Germany; 3 Department of Internal Medicine A, Ernst-Moritz-Arndt University of Greifswald, Greifswald, Germany; 4 Institute of Anatomy and Cell Biology, Ernst-Moritz-Arndt University of Greifswald, Greifswald, Germany; 5 Institute for Community Medicine, Ernst-Moritz-Arndt University of Greifswald, Greifswald, Germany

**Keywords:** eGFR, Insulin-like growth factor, IGF-I, IGFBP-3, Renal function, Study of health in Pomerania

## Abstract

**Background:**

Insulin-like growth factor I (IGF-I), which is mostly carried in blood by IGF-binding protein 3 (IGFBP-3), was associated to the glomerular filtration rate and chronic kidney disease in a multiethnic study among US adults. The aim of the present study was to investigate whether serum IGF-I or IGFBP-3 are associated with estimated glomerular filtration rate (eGFR) in a population-based study of Caucasian adults.

**Methods:**

Data from 4028 subjects (2048 women) aged 20 to 81 years from the Study of Health in Pomerania (SHIP) were analyzed. Total serum IGF-I and IGFBP-3 concentrations were determined by chemiluminescence immunoassays and categorized into sex- and age-specific quartiles.

**Results:**

After adjusting for age, waist circumference and type 2 diabetes mellitus, analysis of variance (ANOVA) revealed inverse associations between serum IGF-I concentrations and eGFR in men as well as between serum IGFBP-3 concentrations and eGFR in men and women. Logistic regression analyses confirmed these findings and showed that high IGF-I or IGFBP-3 concentrations were associated with an increased risk of decreased eGFR (<60 mL/min/1.73 m^2^) in men or women. These relations became stronger when lower eGFR cut-offs were used for the analyses.

**Conclusion:**

Our data revealed associations of increased serum IGF-I concentrations and decreased eGFR in men but not in women and an association of increased serum IGFBP-3 concentrations and decreased eGFR in both sexes.

## Background

The insulin-like growth factor (IGF) system consists of two ligands (IGF-I and IGF-II), three IGF receptors (IGF-I receptor; IGF-II receptor; insulin receptor) and six high-affinity IGF-binding proteins (IGFBPs; IGFBP-1-6) [1]. IGF-I is a polypeptide with a high sequence similarity to insulin and is mainly synthesized in the liver upon stimulation by growth hormone (GH) [1]. In the blood, IGF-I is mostly bound to IGF-binding protein 3 (IGFBP-3) [1]. IGF-I is involved in the regulation of growth and cellular proliferation in humans. Furthermore, IGF-I promotes kidney growth and increases glomerular filtration rate (GFR) as well as renal plasma flow [2]. On the other side, it is widely accepted that kidney disease influence the IGF/GH axis [3]. Serum IGF-I concentrations are associated with arterial hypertension [4,5] and diabetes mellitus [5], which in turn are known risk factors for chronic kidney disease (CKD). With an estimated prevalence of 11%, CKD represents a worldwide public health problem [4,5], which is associated with a poor quality of life [6] and due to renal transplantation and haemodialysis, with intensive costs [7].

Studies on the relation between IGF-I or IGFBP-3 and renal function yielded conflicting results [3,8-10]. Data from patients who were investigated due to proteinuria or haematuria or renal impairment revealed that IGF-I and IGFBP-3 concentrations seem to be independent of renal function [8]. In a small clinical study [3] among patients with renal failure IGF-I was related to a reduced creatinine clearance. A further small study [9] showed that CKD patients had higher serum IGF-I and IGFBP-3 concentrations than healthy individuals suggesting a relation between the IGF/GH axis and CKD. In the population-based National Health and Nutrition Examination Survey (NHANES) III study, including 5388 subjects of different races and ethnicities, increasing serum IGF-I concentrations were associated with a higher risk of CKD [10]. Clear and strong associations between IGF-I and CKD were observed in both sexes with the magnitude of association being stronger in men than in women [10].

The aim of the present study was to investigate the association between serum IGF-I or IGFBP-3 concentrations and the estimated glomerular filtration rate (eGFR) in a Caucasian population. To this end, we used data from the Study of Health in Pomerania (SHIP), including 1980 men and 2048 women.

## Methods

### Study population

SHIP is a population-based cohort study in West Pomerania, a region in northeastern Germany. Details on the SHIP design, recruitment and procedures have been published elsewhere [11]. Baseline data collection started in October 1997 and was finished in March 2001. The initial sample of the baseline examination comprised 4308 (net response: 69%) participants. All participants gave written informed consent. The study conformed to the principles of the Declaration of Helsinki as reflected by an a priori approval of the Ethics Committee of the University of Greifswald.

Of the 4308 participants, 243 subjects with missing values for serum IGF-I, IGFBP-3 or creatinine concentrations were excluded. Furthermore, we excluded one subject with disease of pituitary gland, currently pregnant women and women with uncertainty of current pregnancy (n = 20) and subjects with missing data for selected confounding factors (n = 16). Altogether, the final study population for the present analyses included 4028 subjects (1980 men; 2048 women) aged 20 to 81 years.

### Data collection

Information on age, sex, sociodemographic characteristics and medical histories were assessed by computer- assisted personal interviews. Smoking status was assessed by self-report and categorized in current smoker and non-smoker. Subjects who participated in physical training during summer or winter for at least 1 h a week were classified as being physically active. The definition of type 2 diabetes mellitus was based on self-reported physician’s diagnosis or self-reported use of antidiabetic medication [anatomic, therapeutic, and chemical (ATC) code: A10] during the last 7 days. Hypertension was defined as systolic blood pressure ≥140 mmHg and/or diastolic blood pressure ≥90 mmHg or the self-reported use of antihypertensive medication. Waist circumference (WC) was measured to the nearest 0.1 cm using an inelastic tape midway between the lower rib margin and the iliac crest in the horizontal plane, with the subject standing comfortably with weight distributed evenly on both feet.

Non-fasting blood samples were analyzed immediately or stored at −80°C. Serum creatinine concentrations were determined with the Jaffé method (Hitachi 717; Roche Diagnostics, Mannheim, Germany). The four-variable Modification of Diet in Renal Disease (MDRD) study equation was used for calculation of the eGFR from serum creatinine [eGFR [mL/min/1.73 m^2^ = 186.3 * serum creatinine [mg/dL]^-1.154^ * age [years]^-0.203^ * (0.742 if female)] [12,13]. CKD was defined as an eGFR <60 mL/min/1.73 m^2^, consistent with the definition by National Kidney Foundation Kidney Disease Outcomes Quality Initiative (KDOQI) of CKD ≥ stage 3 [13].

Total serum IGF-I and IGFBP-3 concentrations were determined by automated two-site chemiluminescence immunoassay (Nichols Advantage; Nichols Institute Diagnostica GmbH, Bad Vilbel, Germany). In both assays, biotin-labeled monoclonal antibodies were used as capture, and in a second step acridinium-ester-labeled monoclonal antibodies were used for detection. To determine IGF-I concentration, the samples underwent preanalytical acidification to separate IGF-I from IGFBPs. The analytical sensitivity was 6 ng/mL, the intraassay imprecision within the range of 63–766 ng/mL was 4.8% and interassay imprecision within the range of 62–811 ng/mL was 6.7%. The IGF-I assay was calibrated against the World Health Organization International Reference Reagent 1988, IGF-I 87/518. The analytical sensitivity of the IGFBP-3 assay was 20 ng/mL, the intra- and interassay imprecision within the range of 227–2703 ng/mL were 5.8 and 11%, respectively. The assay reference standard was analytically prepared with glycosylated recombinant human IGFBP-3. Only one lot of reagents was used for all IGFBP-3 measurements. Established reference values in SHIP have been shown elsewhere [14]. Serum IGF-I and IGFBP-3 concentrations were either used as continuous parameters or were categorized into four groups according to the sex- and age-specific quartiles of distribution Additional file 1: Table S1.

### Statistical analyses

Continuous data are expressed as median (25th; 75th quartile); nominal data are expressed as percentage. For bivariate comparisons between women and men the Kruskal-Wallis test (continuous data) or the *χ*^2^-test (nominal data) were used. Analysis of variance (ANOVA) or logistic regression models adjusted for age, waist circumference and type 2 diabetes mellitus were performed to assess the associations between serum IGF-I or IGFBP-3 concentrations and eGFR. Adjusted means and odds ratios (ORs) with 95% confidence intervals (CIs) were calculated. Analyses were performed for the whole cohort and separately for men and women. Additional analyses were performed by using different cut-offs for eGFR (50, 55, and 65 mL/min/1.73 m^2^, respectively). In sensitivity analyses all models were repeated with hypertension as confounder or after the exclusion of subjects with type 2 diabetes mellitus. A p-value < 0.05 was considered statistically significant. Statistical analyses were performed with SAS 9.2 (SAS Institute Inc., Cary, NC).

## Results

The baseline characteristics of our study population demonstrated that men were older, more often smokers, more often affected by hypertension and had lower serum IGFBP-3 concentrations than women (Table 1). Regarding kidney function, men had a higher eGFR and were less often affected by CKD, albeit they had higher serum creatinine concentrations than women.

**Table 1:** Characteristics of the study population

** Characteristics**	**Men**	**Women**	**p**
	**(n = 1980)**	**(n = 2048)**	
Age (years)	52 (37; 65)	49 (36; 62)	<0.01
Waist circumference (cm)	95.2 (87.5; 103.0)	81.5 (72.8; 92.2)	<0.01
Physical activity (%)	41.5	43.7	0.17
Current smoker (%)	33.2	27.2	<0.01
Type 2 diabetes mellitus (%)	8.7	7.4	0.12
Hypertension (%)	62.7	41.7	<0.01
Serum IGF-I (ng/mL)	131 (103; 168)	134 (101; 172)	0.36
Serum IGFBP-3 (ng/mL)	1795 (1473; 2110)	1971 (1666; 2279)	<0.01
Serum creatinine (mg/dL)	1.02 (0.94; 1.12)	0.87 (0.80; 0.94)	<0.01
eGFR (mL/min/1.73m2)	83.02 (73.54; 92.97)	74.84 (66.83; 84.51)	<0.01
CKD (%)	6.6	11.1	<0.01

Continuous data are given as median (25th; 75th quartile); nominal data are given as percentages. χ^2^-test (nominal data) or Kruskal-Wallis test (interval data). *IGF-I* = insulin-like growth factor I; *IGFBP-3* = insulin-like growth factor-binding protein 3; *eGFR* = estimated glomerular filtration rate; *CKD* = chronic kidney disease defined as eGFR < 60 ml/min/1.73 m^2^.

**Figure 1:**
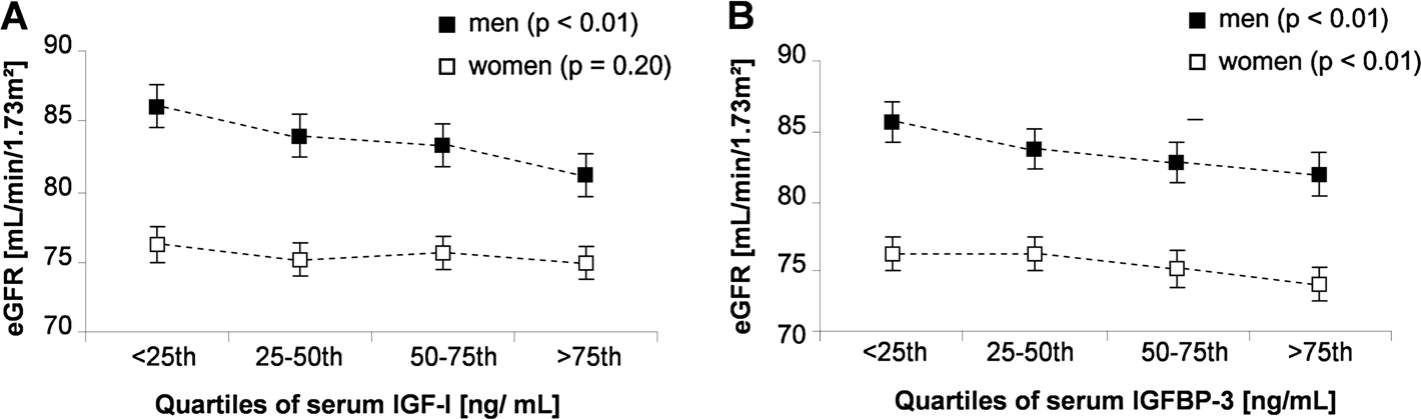
**Mean estimated glomerular filtration rate (eGFR) with 95% confidence interval by (A) insulin-like growth factor I (IGF-I) and (B) IGF-binding protein 3 (IGFBP-3) quartiles in men (*black square*) and women (*white square*).** Analyses of variance were adjusted for age, waist circumference and type 2 diabetes mellitus.

Multivariable ANOVA revealed inverse associations between serum IGF-I or IGFBP-3 and eGFR in men and an inverse association between serum IGF-I and eGFR in women (Figure 1). In detail, in men the eGFR decreased with respect to serum IGF-I concentrations up to 4.9 mL/min/1.73 m^2^ and with respect to serum IGFBP-3 concentrations up to 3.7 ml/min/1.73 m^2^ from the lowest to the highest exposure group. In women the eGFR decreased up to 1.3 ml/min/1.73 m^2^ from the lowest to the highest IGF-I group.

**Figure 2:**
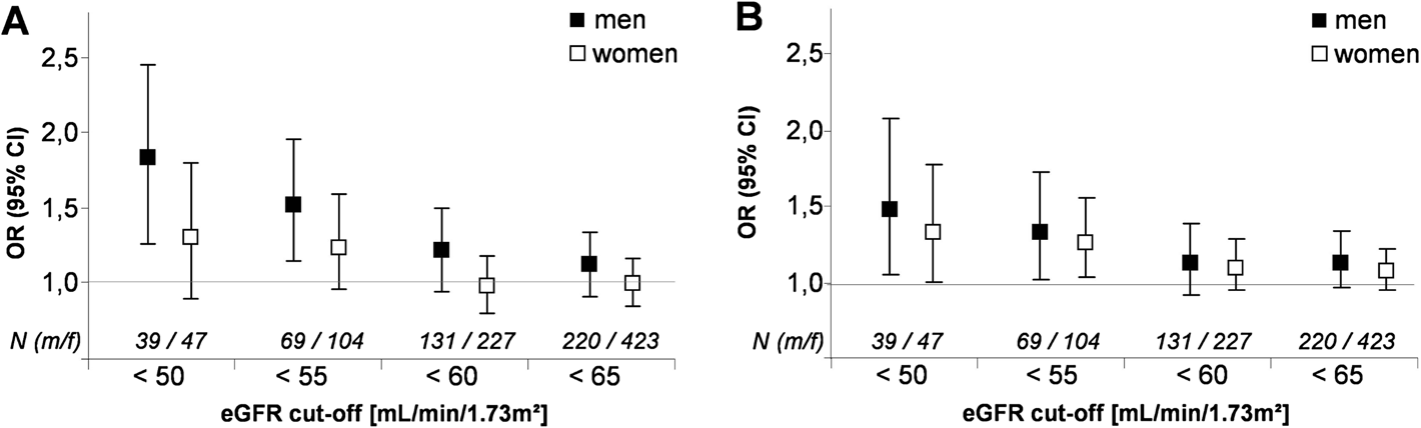
**Odds ratio (OR) with 95% confidence interval for renal dysfunction using different cut-offs for estimated glomerular filtration rate (eGFR) by 1 standard deviation increase in insulin-like growth factor 1 (A) and IGF binding protein 3 (B) in men and women.** N (m/f) = number of participants (m = male; f = female). Logistic regression analyses were adjusted for age, waist circumference and type 2 diabetes mellitus.

To clarify the association between IGF-I or IGFBP-3 and renal dysfunction we used logistic regression models adjusted for age, waist circumference and type 2 diabetes mellitus. Using serum IGF-I or IGFBP-3 concentrations as continuous variables, we observed non-significant associations between increasing concentrations of IGF-I (men: OR for one standard deviation (SD) increase 1.2 [95% CI 0.95-1.50]; women: OR 0.97 [95% CI 0.80-1.19]) or IGFBP-3 (men: OR 1.13 [95% CI 0.93-1.38]; women: OR 1.11 [95% CI 0.96-1.29]) and CKD (Figure 2). However, by varying the cut-off to define low eGFR (<50, <55, <60, <65 mL/min/1.73 m^2^), we observed that the relation between serum IGF-I or IGFBP-3 concentrations and eGFR became significant when lower cut-offs were used in both sexes. In detail, the odds for having an eGFR <50 mL/min/1.73 m^2^ increased with rising IGF-I levels (men: OR per SD increase 1.76 [95% CI 1.27-2.45]; women: 1.27 [95% CI 0.90-1.80]) or IGFBP-3 levels (men: OR per SD increase 1.48 [95% CI 1.06-2.07]; women 1.33 [95% CI 1.01-1.77]).

Our main results were confirmed in sensitivity analyses with hypertension (n = 2095) as confounder or after exclusion of all subjects with type 2 diabetes mellitus (n = 323) (data not shown).

## Discussion

In the present study, we observed an association between increased serum IGF-I and decreased eGFR in men but not in women and between increased serum IGFBP-3 concentrations and decreased eGFR in both sexes. Our findings regarding IGF-I confirm the data from the NHANES III study [10] revealing a positive association between serum IGF-I concentrations and risk of CKD.

A study in monozygotic and dizygotic adults twin pairs of the same sex showed that women had higher serum IGFBP-3 concentrations than men [15]. Furthermore, sex-specific differences in serum IGF-I concentrations were detected and showed that the median serum IGF-I value was 3 ng/mL lower in women compared to men [14,15]. The differences in serum IGF-I or IGFBP-3 concentrations between men and women justify performing sex-segregated analysis tests and may explain the slightly different results with respect to the association between IGF-I and CKD in the present study. However, the positive association between increased serum IGF-I and decreased eGFR only barely missed statistical significance in women. The same trend was observed in NHANES III [10].

In comparison to our results, a small Danish study found lower free serum IGF-I and higher serum IGFBP-3 concentrations in patients with CKD compared to healthy controls [3]. However, total IGF-I concentrations were slightly but not significantly increased in patients with CKD compared to healthy controls. Peritoneal dialysis and haemodialysis patients had significantly higher serum IGF-I and IGFBP-3 concentrations than subjects with normal renal function [16]. Another study [17] showed normal serum IGF-I concentrations, but elevated serum IGFBP-3 concentrations in patients with CKD and different types of renal osteodystrophy compared to healthy control subjects. Taking into account the decreased renal clearance and thus an increased half-life of IGF-I in CKD [18], elevated serum IGF-I concentrations may be expected and normal serum IGF-I concentrations may indicate reduced production. In advanced renal failure, other condition such as metabolic acidosis has been shown to reduce serum IGF-I concentrations [19]. The altered metabolic milieu in severe CKD affects the secretion of hormones and the hormone-induced response of target tissues, causing endocrine dysfunctions [20].

A further study among 500 never-treated hypertensive patients [4] showed an positively and strong association between IGF-I and eGFR which is in contrast to our results even if we have a high proportion of subjects with hypertension. Possible reason for this discrepancy might be that compared to the never-treated hypertensive subjects investigated by Peticone et al. [4] the large part of our participants with hypertension (44.9%) were treated. There is strong evidence for an association of renal function and cardiovascular mortality even in non-dialysis-dependent CKD [21], which was confirmed by a recent large meta-analysis of 21 general population cohorts [22]. This association might be modulated by different IGF-I serum concentrations. In previous epidemiological studies IGF-I, as well as IGFBP-3, has been suggested as risk factor for all-cause and cardiovascular mortality [23]. In a recent meta-analysis [24] low, as well as high, IGF-I serum concentrations were associated with increased mortality in the general population. Thus, in early or moderate renal failure, serum IGF-I concentrations may be elevated due to increased IGF-I half-life and may become clinically relevant in later stages of renal failure. Nevertheless, we assume that the IGF/GH axis is a biomarker rather than a risk factor for renal diseases and reflects the healthy status which might also be influenced by renal disorders. Therefore, IGF-I is not specific for impaired renal function.

IGFBP-3 is specifically cleaved by proteases and low-molecular weight IGFBP-3 fragments accumulating in CKD may be detected by the usual radioimmunoassay. In an earlier study in CKD patients, the intact serum IGFBP-3 concentrations stayed within normal limits [25].

The key strength of our study is the large population-based sample of 4028 men and women aged 20–81 years. A further strength is the high grade of quality assurance of the laboratory method. All assays were performed on a single analyzer using the same reagents and were conducted by skilled technicians and, therefore, minimizing analytical variability. However, a limitation of our study arise from the cross-sectional design that is generally not suitable to prove causal relations. Furthermore, no measurements of free IGF-I or IGF-II were available which would provide a more complete view on the relation between the IGF/GH axis and renal function.

## Conclusion

In conclusion, we found an association of increased serum IGFBP-3 concentrations with decreased eGFR in both sexes and an association of increased serum IGF-I concentrations and decreased eGFR in men but not in women.

## Abbreviations

ANOVA: Analysis of variance; ATC: Anatomic therapeutic and chemical; CI: Confidence interval; CKD: Chronic kidney disease; GANI_MED: Greifswald approach to individualized medicine; eGFR: Estimated glomerular filtration rate; Fig: Figure; GFR: Glomerular filtration rate; h: Hour; IGF-I: Insulin-like growth factor I; IGFBP-3: IGF-binding protein 3; MDRD: Modification of diet in renal disease; NHANES: National health and nutrition examination survey; OR: Odds ratio; SD: Standard derivation; SHIP: Study of Health in Pomerania; Tab: Table; WC: Waist circumference.

## Competing interests

The authors declare that they have no competing interests.

## Authors’ contributions

Conception and Design: KD, NF, HW. Data Analysis: KD. Interpretation of data: All. Article drafting: KD, NF, HW. Final approval: All.

## Pre-publication history

The pre-publication history for this paper can be accessed here:

http://www.biomedcentral.com/1471-2369/13/169/prepub

## Supplementary Material

Additional file 1 **Table S1.** Serum IGF-I and IGFBP-3 distribution by sex and age-group. Click here for file
