# Supplementary material for: Association between serum insulin-like growth factor I or IGF-binding protein 3 and estimated glomerular filtration rate: results of a population-based sample
Source: BMC Nephrol. 2012 Dec 13;13:169. doi: 10.1186/1471-2369-13-169 (PMC3563443; doi:10.1186/1471-2369-13-169)
Supplement: Additional file 1 — Table S1. Serum IGF-I and IGFBP-3 distribution by sex and age-group. [file 1471-2369-13-169-S1.doc]

**Supplemental**

**Table 1**

**Serum IGF-I and IGFBP-3 distribution by sex and age-group**

| Age group | n |  |  | Serum IGF-I (ng/mL) |  |  | Serum IGFBP-3 (ng/mL) |  |
| --- | --- | --- | --- | --- | --- | --- | --- | --- |
| (years) | Men | Women |  | Men | Women |  | Men | Women |
| 20 - 29 | 251 | 279 |  | 198.26 (163.86; 244.04) | 197.77 (159.96; 245.35) |  | 2036.89 (1812.97; 2319.00) | 2167.12 (1914.59; 2522.38) |
| 30 - 39 | 328 | 388 |  | 150.79 (123.74; 185.83) | 154.21 (129.57; 191.42) |  | 1940.21 (1652.37; 2244.53) | 2019.50 (1766.00; 2291.40) |
| 40 - 49 | 331 | 370 |  | 134.74 (110.41; 164.95) | 134.40 (108.22; 164.02) |  | 1951.00 (1625.64; 2238.81) | 1957.00 (1696.00; 2209.60) |
| 50 - 59 | 351 | 396 |  | 119.19 ( 95.64; 149.58) | 127.07 ( 99.66; 156.44) |  | 1760.00 (1455.43; 2052.18) | 1974.00 (1676.33; 2303.00) |
| 60 - 69 | 407 | 353 |  | 117.04 ( 89.59; 142.54) | 105.20 ( 82.17; 134.98) |  | 1607.04 (1322.00; 1913.34) | 1873.00 (1552.48; 2176.36) |
| > 70 | 312 | 262 |  | 107.22 ( 84.87; 134.65) | 97.03 ( 77.03; 124.81) |  | 1476.99 (1164.16; 1771.00) | 1770.16 (1463.00; 2110.23) |

Continuous data are given as median (25th; 75th quartile)
